# Supplementary material for: Co-targeting KRAS and Exportin1 as an effective therapeutic strategy for KRASG12D mutant pancreatic ductal adenocarcinoma
Source: bioRxiv. 2025 Nov 25:2025.11.21.689857. Preprint. [Version 1] doi: 10.1101/2025.11.21.689857 (PMC12697726; doi:10.1101/2025.11.21.689857)
Supplement: Supplement 1 [file media-1.pdf]

Supplementary Data

Co-targeting KRAS and Exportin1 as an effective therapeutic strategy for KRASG12D mutant pancreatic ductal adenocarcinoma

Figure S1.

A. HPAF-II

CI for experimental values:

**MRTX1133** (nM)	**KPT8602** (nM)	**Fa**	**CI**
125	125	0.897751	**0.131**
250	250	0.899346	**0.250**
500	500	0.913224	**0.314**

B. HPAC

CI for experimental values:

**MRTX1133** (nM)	**KPT8602** (nM)	**Fa**	**CI**
125	125	0.585654	**0.359**
250	250	0.670939	**0.126**
500	500	0.625339	**0.625**

C. AsPC-1

CI for experimental values:

**MRTX1133** (nM)	**KPT8602** (nM)	**Fa**	**CI**
125	125	0.528231	**0.614**
250	250	0.580176	**0.619**
500	500	0.60527	**0.904**

D. Panc-1

CI for experimental values:

**MRTX1133** (nM)	**KPT8602** (nM)	**Fa**	**CI**
125	125	0.299984	**0.207**
250	250	0.3048	**0.393**
500	500	0.298897	**0.836**

E. 6694c2

CI for experimental values:

**MRTX1133** (nM)	**KPT8602** (nM)	**Fa**	**CI**
125	125	0.17478	**0.727**
250	250	0.379555	**0.729**
500	500	0.513621	1.101

Figure S1: Synergistic inhibitory effects of KRASG12Di-XPO1i treatments on the growth of several PDAC cell lines. Different dose combinations of MRTX1133 with KPT8602 (Eltanexor) synergistically inhibit the proliferation of KRASG12D mutant PDAC cell lines **(A)** HPAF-II, **(B)** HPAC, **(C)** AsPC-1, **(D)** Panc-1, and **(E)** 6694c2 as indicated by combination index (CI) values less than 1, determined by CalcuSyn**.** All results are expressed as percentage of control ± S.E.M of six replicates.

Figure S2.

A. HPAF-II (3D)

CI for experimental values:

MRTX1133 (nM)	KPT8602 (nM)	Fa	CI
31.25	31.25	0.661561	**0.383**
62.5	62.5	0.742375	**0.382**
125	125	0.833192	**0.287**
250	250	0.858594	**0.405**
500	500	0.896835	**0.427**
1000	1000	0.893408	**0.911**

B. HPAC (3D)

CI for experimental values:

MRTX1133 (nM)	KPT8602 (nM)	Fa	CI
62.5	62.5	0.417725	**0.543**
125	125	0.642479	**0.381**
250	250	0.873315	**0.166**

C. KPC-313 (3D)

CI for experimental values:

MRTX1133 (nM)	KPT8602 (nM)	Fa	CI
31.25	31.25	0.256513	**0.337**
62.5	62.5	0.392396	**0.198**
125	125	0.498429	**0.170**
250	250	0.558221	**0.213**

Figure S2: KRASG12Di synergizes with XPO1i to inhibit the viability of PDAC cell lines in 3D cultures. Synergistic inhibitory effects of the combination treatment at multiple doses of MRTX1133 and KPT8602 (Eltanexor) in constant ratio on the viability of **(A)** HPAF-II, **(B)** HPAC, and **(C)** KPC-313 cells in 3D cultures, as demonstrated by CI < 1 determined by CalcuSyn. All results are expressed as percentage of control ± S.E.M of four replicates.

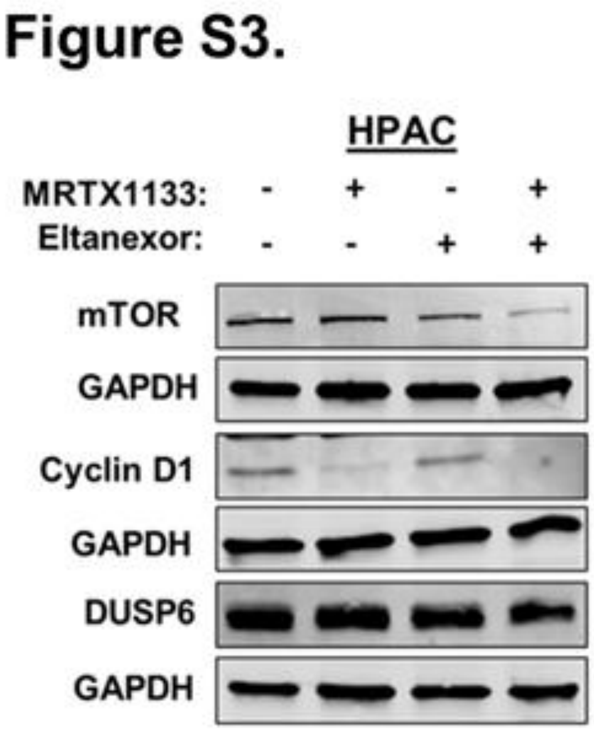

Figure S3: KRASG12Di-XPO1i modulates KRAS signaling and cell cycle. Immunoblot showing reduced expression of mTOR, DUSP6 and Cyclin D1 in combination treated HPAC cells treated with 10 nM and 100 nM of MRTX1133 and Eltanexor, respectively.

Figure S4.

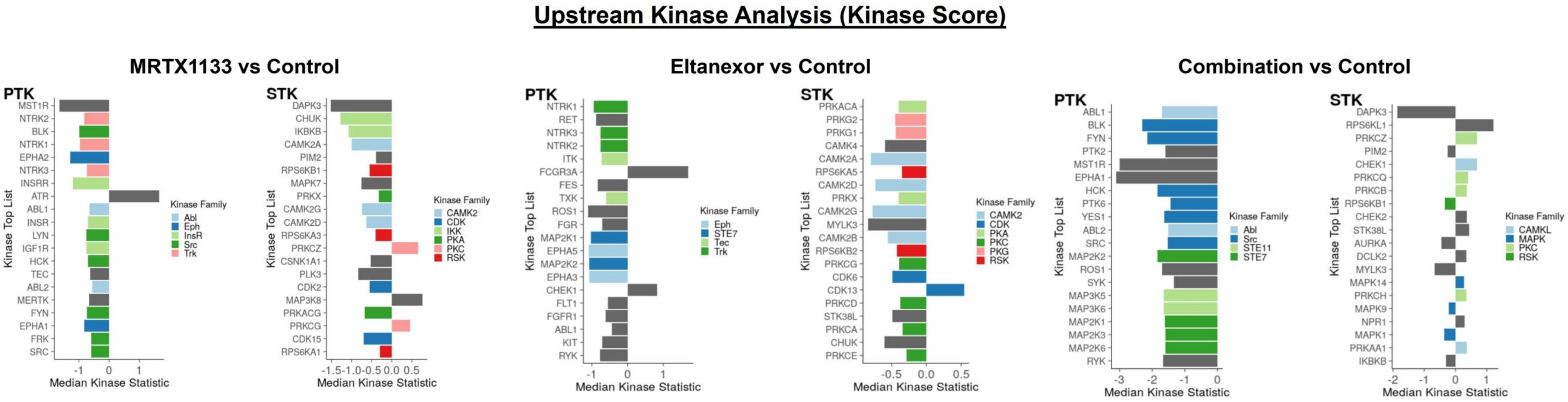

Figure S4: Upstream Kinase Analysis (UKA). Following phosphosite analysis of drug treated PDAC cell lysates, UKA was performed using sets of phosphosites, which were linked to each kinase to predict differential kinase activity. Data shows clear reduction in kinase scores of several kinases associated with MAPK signaling in combination treated HPAC cell lysates.

Figure S5.

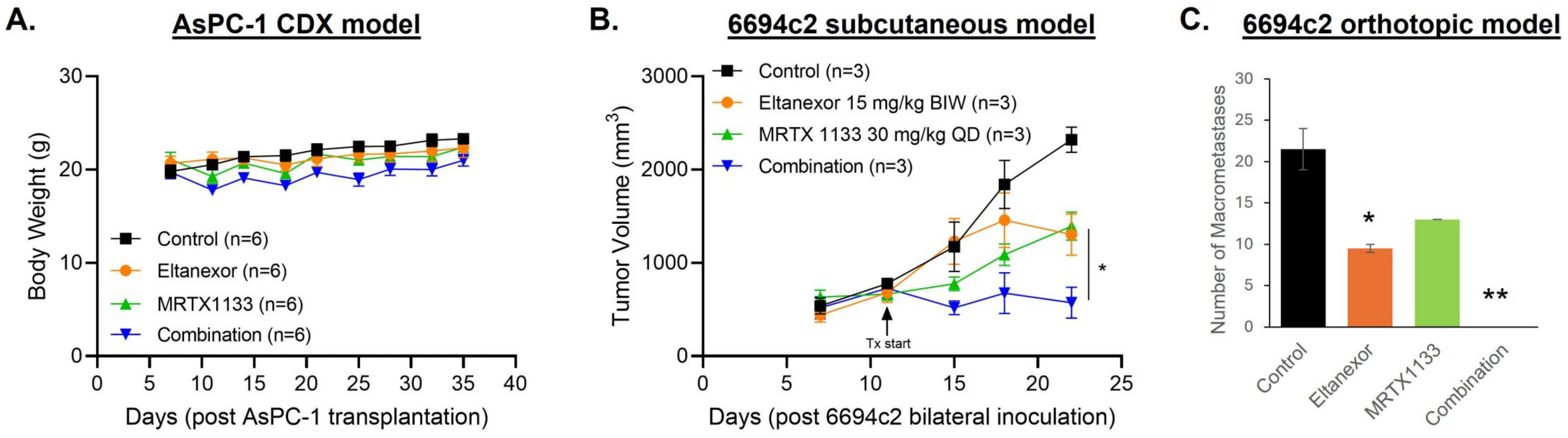

Figure S5: Antitumor and antimetastatic effects of KRASG12Di-XPO1i combination in PDAC *in vivo* models. (A) No substantial loss in the animal body weights was observed in the AsPC-1 CDX tumor bearing ICR-SCID mice treated with MRTX1133, Eltanexor or their combination. **(B)** The combination of MRTX1133 and eltanexor demonstrates significantly (* *P < 0.05)* greater suppression of 6694c2 KPC tumor volumes at the end of treatment compared to either of the single agents alone. **(C)** Barplot showing significant loss of total number of macrometastases in combination treated C57BL/6 mice bearing orthotopic 6694c2 allografts. * P < 0.05, ** P < 0.01 compared to control.

Supplementary Table 1. **Sequences of primers used to perform RT-PCR**

Primers	Sequences	
MAP2K1	Forward	CAATGGCGGTGTGGTGTTC
	Reverse	GATTGCGGGTTTGATCTCCAG
MAP2K2	Forward	AGGTCCTGCACGAATGCAA
	Reverse	CGTCCATGTGTTCCATGCAA
MAP2K3	Forward	GACTCCCGGACCTTCATCAC
	Reverse	GGCCCAGTTCTGAGATGGT
MAP3K5	Forward	CTGCATTTTGGGAAACTCGACT
	Reverse	AAGGTGGTAAAACAAGGACGG
MAP3K6	Forward	GATGCCTTCTACAACGCGGAT
	Reverse	CACGCACACCAAGGTGGTA
MST1R	Forward	CTTTGACGTGAAGTACGTGGT
	Reverse	CGTATGGCTACAAACACAGCAC
ABL1	Forward	TGAAAAGCTCCGGGTCTTAGG
	Reverse	TTGACTGGCGTGATGTAGTTG
ABL2	Forward	GTTGAACCCCAGGCACTAAAT
	Reverse	CAACGAAGAGATTAGGGTCACTC
ROS1	Forward	CCACATAATCTGAGTGAACCGTG
	Reverse	CGCTGCTACAGCCAACCTC
SYK	Forward	CATGGAAAAATCTCTCGGGAAGA
	Reverse	GTCGATGCGATAGTGCAGCA
β-actin	Forward	GCACAGAGCCTCGCCTT
	Reverse	TCATCATCCATGGTGAGCTG

Supplementary Table 2. **Phosphosite analysis.** Number of differentially phosphorylated phosphosites (PTK and STK) between conditions are identified using ANOVA-Dunnett's test.

Assay Type	PTK		STK	
**Comparisons**	Up	Down	Up	Down
**MRTX1133 vs Control***	4	13	0	4
**Eltanexor vs Control***	1	3	0	1
**Combination vs Control***	0	24	1	6
*Significance was obtained using one-way ANOVA followed by post-hoc Dunnett's test. $p<0.05$				
